# Unveiling spatial patterns of West Nile virus emergence in northern Greece, 2010–2023

**DOI:** 10.1016/j.onehlt.2024.100888

**Published:** 2024-09-05

**Authors:** Anastasia Angelou, Lea Schuh, Nikolaos I. Stilianakis, Spiros Mourelatos, Ioannis Kioutsioukis

**Affiliations:** a Department of Physics, University of Patras, Greece; b European Commission, Joint Research Centre (JRC), Ispra, Italy; c Department of Biometry and Epidemiology, University of Erlangen-Nuremberg, Erlangen, Germany; d Ecodevelopment S.A., Thessaloniki, Greece

**Keywords:** West Nile virus human cases, Mosquito abundance, Infected mosquitoes, Spatial autocorrelation, Moran's I, Climatic factors

## Abstract

The Region of Central Macedonia (RCM) in Northern Greece recorded the highest number of human West Nile virus (WNV) infections in Greece, despite considerable local mosquito control actions. We examined spatial patterns and associations of mosquito levels, infected mosquito levels, and WNV human cases (WNVhc) across the municipalities of this region over the period 2010–2023 and linked it with climatic characteristics. We combined novel entomological and available epidemiological and climate data for the RCM, aggregated at the municipality level and used Local and Global Moran's I index to assess spatial associations of mosquito levels, infected mosquito levels, and WNVhc. We identified areas with strong interdependencies between adjacent municipalities in the Western part of the region. Furthermore, we employed a Generalized Linear Mixed Model to first, identify the factors driving the observed levels of mosquitoes, infected mosquitoes and WNVhc and second, estimate the influence of climatic features on the observed levels. This modeling approach indicates a strong dependence of the mosquito levels on the temperatures in winter and spring and the total precipitation in early spring, while virus circulation relies on the temperatures of late spring and summer. Our findings highlight the significant influence of climatic factors on mosquito populations (∼60 % explained variance) and the incidence of WNV human cases (∼40 % explained variance), while the unexplained ∼40 % of the variance suggests that targeted interventions and enhanced surveillance in identified hot-spots can enhance public health response.

## Highlights

• The entomological and epidemiological “hot-spot” of the region is identified.
• Warm winter and spring and wet March cause an increase in the mosquito abundances.
• Higher temperatures in May and summer lead to an elevated number of human cases.
• Environmental factors account for over 40 % (60 %) of the WNVhc (MCPL) variability.
• By including last year's WNV circulation data, prediction accuracy increases by 13 %.

## 1 Introduction

West Nile virus (WNV) is a neurotropic virus belonging to the *Flavivirus* genus. The virus is maintained in a mosquito-bird-mosquito transmission cycle, where infected mosquitoes, predominantly *Culex pipiens* [1,2], transmit WNV to birds [3] and vice versa [4]. Once infected mosquitoes feed on humans or other mammals, WNV is transmitted [5,6]. However, the low level of viremia is not sufficient for viral transmission from humans to mosquitoes, rendering the human a dead-end host for WNV [7]. Upon infection, about 20 % of humans develop West Nile fever (WNF) and less than 1 % develop West Nile neuro-invasive disease (WNND), which can be lethal [8]. To date, there is no vaccine against WNV [9] and disease prevention is focusing on mosquito control and personal protective measures to restrict exposure to infected mosquitoes. Until the end of the 1990s, WNV was considered a minor risk for humans because of its sporadic occurrence [10]. However, over the last two decades there have been repeated local outbreaks across various regions worldwide, such as Europe, Africa, Asia, United States [[11], [12], [13], [14], [15]]. The WNV outbreak in Romania in 1996 with 393 WNVhc [16] and the detection of the virus in the Americas in late summer 1999 were messengers of the disease re-emergence [17]. In Greece, WNVhc were first recorded in 2010 (262 cases, 35 deaths), while two major WNV outbreaks occurred during the period 2010–2023 [18]: in 2018, 316 laboratory-confirmed WNVhc (50 deaths) were reported, and in 2022, 286 WNVhc were identified (32 deaths). Overall, Greece reported the highest number in WNVhc in 2022 in Europe, after Italy [19,20], where most WNVhc occurred in RCM in Northern Greece. Identification of areas of high WNV transmission and disease risk is highly relevant for disease prevention measures such as mosquito control activities [21]. However, observed fluctuations and occasional explosive numbers of cases in some years, render WNV risk mapping challenging [22].

Various studies, conducted across different locations and years, consistently demonstrated pronounced spatial clustering of *Culex* pool counts [23,24] or WNV infection in humans [[24], [25], [26], [27], [28], [29]] and provide extensive analyses of the environmental and climatic conditions related to mosquito abundance and consequently also WNVhc [[30], [31], [32], [33], [34]]. Particularly, higher temperatures during spring and summer were identified to promote WNVhc [32,[35], [36], [37], [38]], while rainfall, humidity, soil water content, and wind were reported to decrease mosquito abundance and the recorded number of WNVhc [35,[37], [38], [39]].

Utilizing a novel entomological and epidemiological dataset in the RCM during the 2010–2023 period, upscaled at municipality level (below NUTS3), we investigate the spatial distributions of mosquito levels, infected mosquito levels and WNVhc through the Moran's I indices to identify the “hot-spot” areas common at all three maps. Then, using climatic data from Copernicus aggregated at municipality and monthly-seasonal scale, we fit a Generalized Linear Mixed Model to identify the factors driving the observed mosquito levels, infected mosquito levels and WNVhc and ultimately quantify the fraction of the observed variability explained by only environmental factors, under the current state of surveillance and targeted actions.

## 2 Materials and methods

### 2.1 Data collection and pre-processing

#### 2.1.1 The region of Central Macedonia

**Fig. 1:**
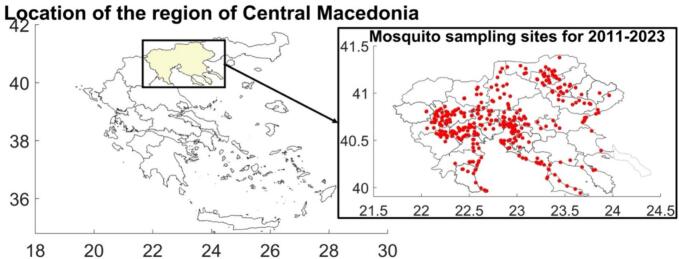
Position of the Region of Central Macedonia in Greece (left) and geographical distribution of the location of mosquito traps (red points) in the region (right). (For interpretation of the references to colour in this figure legend, the reader is referred to the web version of this article.)

Our study focused on the RCM in Northern Greece (Fig. 1**(left)**) consisting of 38 municipalities, examining data at the municipal level. We obtained data on the local administrative division, land use, and area at the municipal level from the Hellenic Statistical Authority (HSA). Detailed information is available in **S1 Appendix**.

#### 2.1.2 Mosquito network

As part of a regional WNV surveillance program, CO_2_ mosquito traps were placed throughout the RCM (Fig. 1**(right)**), positioned based on criteria such as proximity to human settlements, protection from direct sunlight and wind, and historical WNV detection [40]. Some mosquito samples were tested for WNV presence using RT-nested PCR in reference laboratories [19], with testing frequency varying from 0 to 100 % monthly. More information is presented in **S2 Appendix**.

#### 2.1.3 Measure of Culex population level (MCPL)

We calculated the mean monthly *Culex* mosquito abundance from all traps within the same municipality, provided a minimum of two samples were available to avoid spurious peaks. Το reduce the data variability, we defined the *Measure of Culex Population Level* (MCPL) (mosquitoes/km^2^) as the average mosquito abundance of the month during which the maximum number of mosquitoes were recorded, the month directly preceding and following. We defined the mosquito's flight range as 500 m, often reported for *Culex* mosquitoes [41,42]. Only municipalities with 13 years (2011−2023) of mosquito measurements were included to ensure reliability.

#### 2.1.4 Measure of infected mosquito samplings (MIMS)

Based on the average monthly mosquito abundance from all traps within a municipality, we calculated the monthly percentage of infected mosquito samples out of those tested. We defined the *Measure of Infected Mosquito Samplings* (MIMS) as the annual maximum percentage (%) of infected mosquito samples.

#### 2.1.5 WNV human cases (WNVhc)

We obtained laboratory-confirmed WNVhc in RCM from the National Public Health Organisation (EODY), excluding cases with unknown exposure locations or dates. We calculated WNVhc per 100,000 population (WNVhcP100TH) using municipal population data. Reported WNVhc were assumed to represent the actual number, despite underreporting due to asymptomatic cases [43]. Detailed information is available in **S3 Appendix.**

#### 2.1.6 Climatic data

Air temperature and precipitation were considered crucial climatic factors in WNV transmission [44]. Six-hourly air temperature data for RCM from 2010 to 2023 from ERA5 were used to calculate average daily, monthly and seasonal temperatures, and Growing Degree Days at specific month (GDD_MM_). GDD_MM_ was defined as the number of days from January 1 until the end of month MM when the average daily temperature exceeded 14.3 °C, the threshold for virus development [45]. Daily precipitation data were also acquired from ERA5, from which total monthly and seasonal precipitation were calculated.

### 2.2 The local and global Moran index

We computed the Global Moran Index for the median values of mosquito abundance, mosquito positivity, and WNVhc each year (detailed in **S4 Appendix**). The index ranges from −1 to 1, with values near zero indicating no spatial autocorrelation. The Local Moran's Index enabled us to identify clusters (i.e. groups of municipalities) of high values (Hot-spots), revealing significant spatial patterns of WNV activity. The central location(s) within a cluster, i.e. those exhibiting high values and also having neighboring municipalities with similarly high values, form the core of a cluster.

### 2.3 Generalized linear mixed model

We fitted a Generalized Linear Mixed Model (GLMM) to analyze entomological and epidemiological data, considering fixed effects such as environmental factors and random effects attributed to municipalities. We investigated 47 predictor variables: monthly temperature and monthly total precipitation from January to August (16), seasonal temperature and seasonal total precipitation (8), maximum and minimum seasonal temperature (8), GDD from January to August (8), percentages of arable land, heterogeneous agricultural land, and inland wetlands, and the sum of these areas (defined as AAW) (4), and previous year's MCPL, MIMS, and WNVhcP100TH (3) to estimate their influence on MCPL, MIMS, and WNVhcP100TH (predicted variables). The study period was 2011–2023, excluding years with missing data. We ranked models by Marginal and Conditional R-squared (MR^2^ and CR^2^) and selected the best as the Poisson model with a log link function and different predictor for each outcome variable.

Leave one-year-out cross validation was also used for verification and identification of anomalous years. To evaluate the predictive ability of the GLMM, we calculated the Normalized Mean Absolute Error (NMAE) (MAE divided by the mean of the observed data). $\mathrm{MAE}=\frac{1}{n}\sum_{1}^{n}\left|P_{i}-O_{i}\right|,$where n represents the total number of observations, *P*_*i*_ the predicted value, and *O*_*i*_ the actual value of the i^th^ observation (detailed in **S5 Appendix**).

## 3 Results

In this section, we analyzed the spatial distributions of MCPL, MIMS and WNVhc and we fitted a GLMM to identify the factors influencing these levels.

### 3.1 Spatial patterns

#### 3.1.1 Mosquito levels

**Fig. 2:**
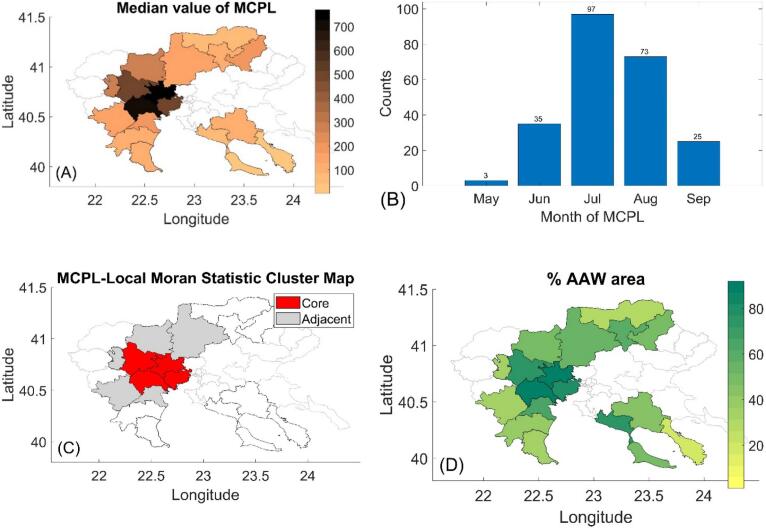
(Α) Map of the median MCPL for each of the 18 municipalities of interest of the RCM for the period 2011–2023. The municipalities excluded from this study are highlighted in gray. (B) Counts of months with the highest annual MCPL across the 18 municipalities of the RCM for the period 2011–2023. (C) Local Moran's I cluster map showing spatial association of median MCPLs with 4 “spatial autocorrelation hot-spot” municipalities: Alexandria, Delta, Pella, and Halkidona. (D) Map of the percentage of AAW areas for each municipality.

We calculated the median MCPL values for each of the 18 municipalities of interest (Fig. 2A). In 22 % (4 out of 18) of the municipalities, the median MCPL exceeded 400 mosquitoes/km^2^. Considering all 18 municipalities across 13 years of observations (234 counts), the annual MCPLs peaked in July (41 %, 97 out of 234) and August (31 %, 73 out of 234) (Fig. 2B). Using the Local Moran Index, we identified 4 “spatial autocorrelation hot-spot” municipalities with significant spatial autocorrelation with adjacent municipalities, located in the Western part (Fig. 2C), overlapping with areas of high median MCPLs. Spatial autocorrelations were significant throughout the study period at a 0.01 significance level, except for 2021 and 2019, indicating consistent clustering of median MCPLs (**Table S5**). Interestingly, the AAW areas per municipality (Fig. 2D) corresponds well with the recorded median MCPLs, suggesting that AAW areas potentially foster increased mosquito abundances (Pearson's correlation coefficient = 0.76, *p*-value<0.001).

#### 3.1.2 Infected mosquito levels

**Fig. 3:**
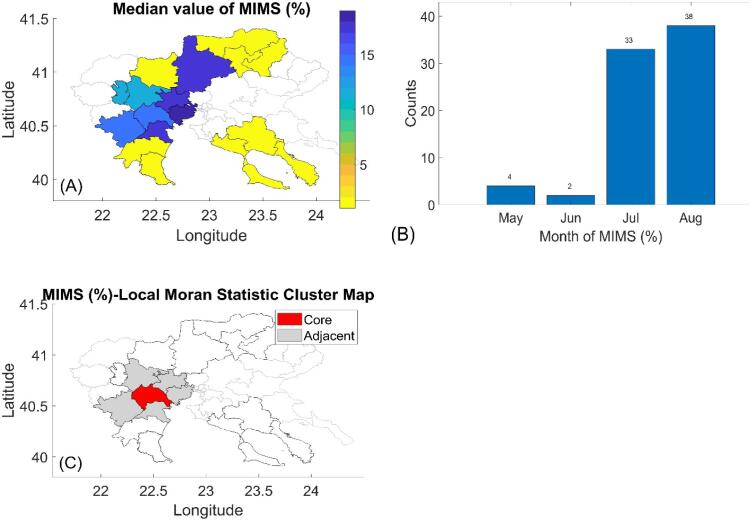
(A) Map of the median MIMS for each of the 18 municipalities of interest in the RCM for the period 2011–2023 (excl. 2014–2017). The municipalities excluded from this study are highlighted in gray. (B) Counts of months of MIMS for the 8 municipalities and 9 years in which infected mosquitos were sampled. (C) Local Moran's I cluster map showing spatial association of median MIMS with one “spatial autocorrelation hot-spot” municipality: Alexandria (in red). (For interpretation of the references to colour in this figure legend, the reader is referred to the web version of this article.)

We investigated WNV circulation by assessing infected mosquito levels through MIMS. The period 2014–2017 was excluded due to the low number of mosquitoes tested and the absence of WNV infection. In 44 % (8 out of 18) of municipalities, the MIMS exceeded 10, while in the remaining 56 %, no infected mosquitoes were found (Fig. 3A), suggesting a bimodality across the RCM. Considering the 8 municipalities and the 9 years of samplings (72 counts), MIMS was primarily recorded in July and August with 46 % (33 out of 72) and 53 % (38 out of 72), respectively (Fig. 3B), matching the months of highest MCPLs. Using the Local Moran Index, we identified one municipality as a “spatial autocorrelation hot-spot”, showing significant spatial autocorrelation with its five adjacent municipalities (Fig. 3C). However, autocorrelation was significant in only 1 out of 9 years at a 0.01 significance level, indicating lower spatial interconnectedness for MIMS compared to MCPL (**Table S5**).

#### 3.1.3 Infected human levels

**Fig. 4:**
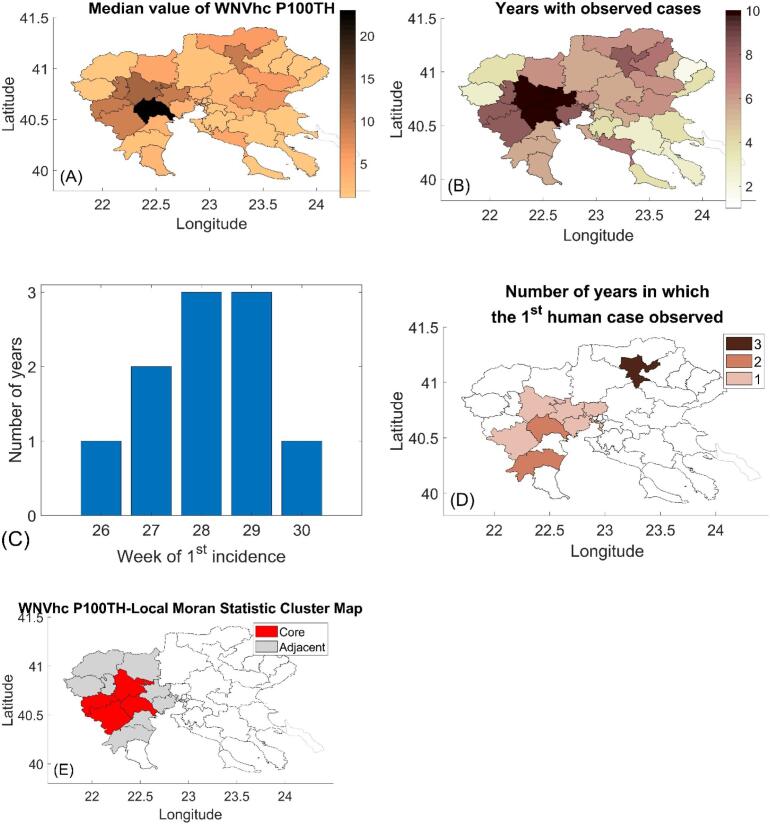
(A) Map of the median WNVhcP100TH in each municipality for the period 2011–2023 (excl. 2014–2017). (B) Geographical distribution of the number of years per municipality (1–10 years) with at least one recorded WNVhcP100TH during the period 2010–2023. (C) Number of years in which the first WNVhc occurred each week. (D) Map of the number of years (1–3 years) in which the first case was recorded per municipality. (E) Local Moran's I cluster map showing spatial associations with 4 “spatial autocorrelation hot-spot” municipalities: Alexandria, Veria, Naousa, Pella. The map concerns the association of the median value of WNVhcP100TH the period 2010–2023 (excl. 2014–2017).

We calculated the medians of laboratory-confirmed WNVhcP100TH per municipality (Fig. 4A). One municipality recorded a particularly high median of 23 WNVhcP100TH. Overall, 26 of the 38 municipalities (68 %) had at least a median WNVhcP100TH of one. Each municipality recorded at least 1 WNVhc from 2010 to 2023 (Fig. 4B), while 18 municipalities (47 %) recorded WNVhc of at least one in more than 5 of the 10 years, demonstrating WNV endemicity in the RCM. Only 3 municipalities (8 %) in the Western region demonstrated high median WNVhcP100TH above 1 for all 10 years, indicating continuous high virus circulation. The first WNVhc were recorded between the 26th and 30th week, corresponding to late of June to early August (Fig. 4C), mostly in the Western RCM (Fig. 4D). In 6 of the 10 years, the first WNVhc was recorded in the 28th or 29th week, corresponding to middle of July. Using the Local Moran Index, we identified a “spatial autocorrelation hot-spot” cluster of four municipalities in the Western RCM with significant spatial autocorrelation with adjacent municipalities for WNVhcP100TH (Fig. 4E). However, significant autocorrelation was observed in only 4 out of 9 years at a 0.01 significance level (**Table S5**).

### 3.2 Spatial predictability

We combined the entomological and epidemiological datasets with climate and Land Use - Land Cover (LULC) data in a GLMM framework. Our findings (Table 1) indicated that a wet March combined with warm winter and spring in municipalities with a high proportion of AAW areas, are expected to cause a heightened MCPL (MR^2^ = 0.61), while the inclusion of a spatial random effect yields a CR^2^ of 0.99. Furthermore, an increased number of GDD_05_ combined with a warm January and summer, and a notable number of WNVhcP100TH in the previous year, are expected to lead to a rise in maximum mosquito positivity (MR^2^ = 0.57, CR^2^ = 0.95). Finally, a wet summer and March combined with a hot summer and May, a wet April, a high number of WNVhcP100TH in the previous year, and a high proportion of suitable LULC are expected to cause an increase in WNVhcP100TH (MR^2^ = 0.53, CR^2^ = 0.89). Therefore, according to the MR^2^, 53–61 % of the observed variability of MCPL, MIMS, and WNVhcP100TH could be explained by the selected inputs, while the remaining variability is accounted by unknown factors. The predictor variables employed at each GLMM had either no significant multicollinearity (MCPL) or moderate multicollinearity (MIMS, WNVhcP100TH) according to their variance inflation factor (VIF) [46]. The VIF of the selected predictor variables for MCPL, MIMS, and WNVhcP100TH were below 2, 2, and 4 respectively (medians: 1.14, 1.25, and 2.92).

**Table 1:** The predictor variables employed in the GLMMs for MCPL, MIMS and WNVhcP100TH. The estimates are presented as incidence rate ratios (IRR), which are exponentiated values from the Poisson model. A 95 % confidence interval (CI) was used in all calculations.

MCPL				MIMS				WNVhcP100TH			
Predictors	Estimate	CI	p-value	Predictors	Estimate	CI	p-value	Predictors	Estimate	CI	p-value
(Intercept)	190.41	89.73–404.1	<0.001	(Intercept)	1.75	0.35–8.81	0.04	(Intercept)	0	0.00–0.00	<0.001
T_winter_	0.89	0.88–0.90	<0.001	T_January_	0.82	0.80–0.85	<0.001	TP_March_	1.15	1.13–1.16	<0.001
TP_March_	1.03	1.02–1.03	<0.001	GDD_05_	0.91	0.90–0.92	<0.001	TP_April_	0.94	0.92–0.95	<0.001
								T_May_	0.58	0.53–0.64	<0.001
T_spring_	0.93	0.92–0.93	<0.001	T_summer_	1.33	1.26–1.41	<0.001	T_summer_	2.29	2.02–2.60	<0.001
								TP_summer_	1.06	1.05–1.06	<0.001
AAW areas	1.03	1.01–1.04	<0.001	WNVhcP100TH previous year	1	0.99–1.00	0.005	WNVhcP100TH previous year	0.99	0.98–0.99	<0.001
								AAW areas	1.02	1.01–1.03	0.002
Marginal R^2^ / Conditional R^2^	**0.61/0.99**			Marginal R^2^ / Conditional R^2^	**0.57/0.95**			Marginal R^2^ / Conditional R^2^	**0.53/0.89**		

**Fig. 5:**
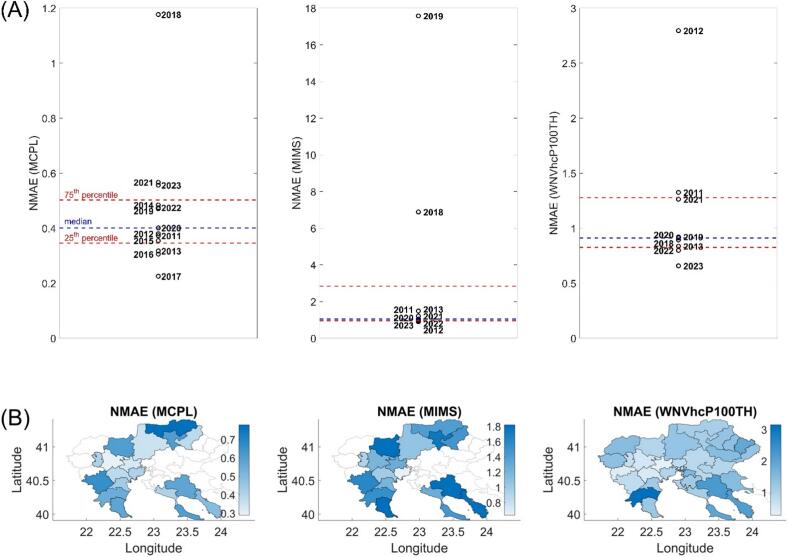
(A) Comparison of MAE values across test years for MCPL, MIMS and WNVhcP100TH. The red dashed lines indicate the 25th and 75th percentiles, while the blue dashed line represents the median MAE value. Each year corresponds to the test set each time where all other years were used for training. (B) Geographical distribution of normalized mean absolute error (NMAE) between predicted and observed MCPL, MIMS, and WNVhcP100TH. The NMAE at each municipality corresponds to the median between all-years through a leave one-year-out cross validation approach. (For interpretation of the references to colour in this figure legend, the reader is referred to the web version of this article.)

Using one-year-out cross-validation, we calculated the NΜΑΕ between predicted and observed MCPL, MIMS and WNVhcP100TH (Fig. 5A). For the entomological variable MCPL, the median NMAE was 0.4 and the inter-quartile range was 0.15, while only 2018 classified as outlier. For the epidemiological variables MIMS and WNVhcP100TH, the median NMAE was on the order of 0.91, with 2018, 2019 and 2012 classified as outliers, respectively. Outliers could be overall justified by the anomalous-low values of the variables in these years relative to the rest (**Fig. S6**).

Excluding the epidemiological inputs in the GLMMs and relying only in the emerged environmental variables (climatic indices and land use), we found M*R*^2^ of 0.51 and 0.40 for MIMS and WNVhcP100TH, respectively. Thus, a considerable fraction of the epidemiological variability on the order of 40–50 % could be attributed to environmental factors alone highlighting their significant impact on mosquito-borne disease dynamics.

Fig. 5B showed the spatial distribution of the NMAE for MCPL, MIMS, and WNVhcP100TH. Focusing on the cores of the “hot-spot” areas in the Western region, the NMAE for MCPL varied from 0.29 to 0.39. The NMAE for MIMS was 1.03, highlighting higher prediction errors compared to MCPL. For WNVhcP100TH, NMAE ranged from 0.36 to 0.74, intermediate values in relation to MCPL and MIMS, indicating reliable predictions in this critical area.

## 4 Discussion

In this study, we used epidemiological and entomological data to investigate the spatial variability in the mosquito populations, the infected mosquito population, and human incidences in the RCM, an area of repeated WNV outbreaks. Therefore, employing the available entomological and epidemiological data of the period 2010–2023 at the municipality level, we investigated statistical associations that could provide insights on the factors involved on WNV spread.

In the RCM, at least one WNVhc was observed in all municipalities (except for the years 2014–2017), demonstrating the high endemicity of WNV. The analysis uncovered spatial patterns of increased WNVhc in the Western part, which may be due to the specific environmental conditions and/or the land cover and use. This region is characterized by water-intensive agricultural cultivation of products such as rice fields, which allow for conditions particularly prone to mosquito breeding and therefore increased risk of virus transmission.

Previous studies identified the Global [25,28] and the Local [23,24] Moran's I index as a useful tool to study spatial dependencies. In our study, the Global Moran's I of the MCPL is statistically significant in 85 % of years (all but 2019 and 2021), as expected since it is a climate dependent parameter and climate variables are spatially correlated. For the mosquitoes positivity the Global Moran's I was statistically significant only in 2020, while for annual WNVhc in four years, namely 2010, 2018, 2020 and 2021. The existence of spatial trends in annual WNVhc could be possibly explained by patterns of bird migration, but there are no data for the RCM to address this assumption.

Via the Local Moran cluster map, the municipality of Alexandria and its surrounding municipalities located in the Western part of the region were identified as a “spatial autocorrelation hot-spot” of the RCM. This is on line with the reported land usage of the municipality, which is mostly covered by drainage canals, rice fields and permanently irrigated land, ideal conditions for the development of mosquitoes. To better understand the identified patterns, a longer time series of entomological data for more municipalities is necessary.

Our modeling analysis highlights the impact of environmental factors on *Culex* population and WNV transmission, with the ability to predict 61 %, 51 % and 40 % of mosquito levels, infected mosquito levels, and WNVhc, respectively, using only climate features. This modeling approach indicates a strong dependence of the mosquito levels on the temperatures in winter and spring and the total precipitation in early spring, while virus circulation relies on the temperatures of late spring and summer. Many studies underscore the critical role of temperature [32,[35], [36], [37], [38],^47^] and precipitation [35,[37], [38], [39],^48^] as major predictors in mosquito development and virus transmission, supporting our findings. Incorporating previous year's virus circulation data enhances the predictive accuracy of the infected humans by 13 %, demonstrating the added value of historical epidemiological data in managing WNV risk. However, various other environmental and non-environmental factors may also influence virus transmission as still 40 % of the observed variability is accounted by unknown factors, highlighting the need for enhanced surveillance.

Overall, prediction errors have smaller year-to-year spread for mosquito abundance and WNVhc compared to mosquito infectivity, probably linked to the data quality in the latter case. Spatially, the median NMAE was 0.47, 1.41 and 1.1 for mosquito levels, infected mosquito levels, and WNVhc respectively, with lower median values at the core municipalities. Taken together the temporal and spatial errors, it is evident that although infected mosquitoes affect the causal pathway between mosquito abundance and infected humans, in their current form (MIMS) are of limited importance for public health.

Our research limitations include the lack of information for larviciding and other spraying actions to control mosquito population and WNV circulation. The discrepancies in the temporal and spatial patterns highlight the uncertainties arising from the use of small sample sizes.

The “spatially autocorrelated hot-spot” municipalities for MCPL, MIMS and WNVhc coincide, marking the expected causal pathway, while they possess increased predictability. The unexplained 40 % variance on the examined variables highlights the need for additional inputs, including larvae data (important for MCPL) and virus circulation information (important for WNVhc). The latter may include for example explicit information for culex positivity or proxy indicators for sentinel positivity.

## 5 Conclusion

Our research highlights the critical role of climate in shaping mosquito populations and WNV transmission in Central Macedonia. The identified spatial interdependencies, particularly in the Western part of the region, along with the strong influence of temperature and precipitation on mosquitoes and infected human levels, demonstrate the importance of climate-driven models for public health. The unexplained variance in WNV incidence suggests that targeted interventions, enhanced surveillance, and consideration of additional factors related to spillover processes are essential for improving WNV risk management and response.

## Funding

This research has been co-financed by the European Regional Development Fund of the European Union and Greek national funds through the Operational Program Competitiveness, Entrepreneurship and Innovation, under the call RESEARCH – CREATE – INNOVATE (project code: Τ2ΕΔΚ-02070). This work was also co-financed from the EIC Horizon Prize “Early Warning for Epidemics”.

## CRediT authorship contribution statement

**Anastasia Angelou:** Writing – original draft, Visualization, Validation, Software, Investigation, Formal analysis. **Lea Schuh:** Writing – review & editing, Methodology. **Nikolaos I. Stilianakis:** Writing – review & editing, Methodology. **Spiros Mourelatos:** Data curation. **Ioannis Kioutsioukis:** Writing – review & editing, Supervision, Resources, Project administration, Methodology, Funding acquisition, Conceptualization.

## Declaration of competing interest

The authors declare that they have no known competing financial interests or personal relationships that could have appeared to influence the work reported in this paper.

## Data Availability

The mosquito data used were provided from EcoDevelopmet S.A. under license, and so are not publicly available. Data are available from the authors upon reasonable request and with permission of EcoDev

## Appendix A Supplementary data

Supplementary material. Image 1

## References

[1] Gray T.J., Webb C.E. (2014). A review of the epidemiological and clinical aspects of West Nile virus. Int J Gen Med.

[2] Marini G., Poletti P., Giacobini M., Pugliese A., Merler S., Rosa R. (2016). The role of climatic and density dependent factors in shaping mosquito population dynamics: the case of *culex pipiens* in northwestern Italy. PLoS One.

[3] Valiakos G., Touloudi A., Iacovakis C., Athanasiou L., Birtsas P., Spyrou V. (2011). Molecular detection and phylogenetic analysis of West Nile virus lineage 2 in sedentary wild birds (Eurasian magpie), Greece, 2010. Euro Surveill..

[4] Rochlin I., Faraji A., Healy K., Andreadis T.G. (2019). West Nile virus mosquito vectors in North America. J. Med. Entomol..

[5] Komar N. (2000). West Nile viral encephalitis. Rev. Sci. Tech..

[6] Colpitts T.M., Conway M.J., Montogomery R.R., Fikrig E. (2012). West Nile virus: biology, transmission, and human infection. Clin. Microbiol. Rev..

[7] Calistri P., Giovannini A., Hubalek Z., Ionescu A., Monaco F., Savini G. (2010). Epidemiology of West Nile in Europe and in the Mediterranean basin. Open Virol J.

[8] Ladbury G.A., Gavana M., Danis K., Papa A., Papamichail D., Mourelatos S. (2013). Population seroprevalence study after a West Nile virus lineage 2 epidemic, Greece, 2010. PLoS One.

[9] World Health Organization (2024). Vector-borne diseases. https://www.who.int/news-room/fact-sheets/detail/vector-borne-diseases.

[10] Dauphin G., Zientara S. (2007). West Nile virus: recent trends in diagnosis and vaccine development. Vaccine.

[11] Kramer L.D., Styer L.M., Ebel G.D. (2008). A global perspective on the epidemiology of West Nile virus. Annu. Rev. Entomol..

[12] Papa A., Xanthopoulou K., Gewehr S., Mourelatos S. (2011). Detection of West Nile virus lineage 2 in mosquitoes during a human outbreak in Greece. Clin. Microbiol. Infect..

[13] Papa A., Xanthopoulou K., Tsioka A., Kalaitzopoulou S., Mourelatos S. (2013). West Nile virus in mosquitoes in Greece. Parasitol. Res..

[14] Petersen L., Brault A., Nasci R. (2013). West Nile virus: review of the literature. JAMA.

[15] Young J., Haussig J., Aberle S., Pervanidou D., Riccardo F., Sekulić N. (2021). Epidemiology of human West Nile virus infections in the European Union and European Union enlargement countries, 2010 to 2018. Euro Surveill..

[16] Tsai T.F., Popovici F., Cernescu C., Campbell G.L., Nedelcu N.I. (1998). West Nile encephalitis epidemic in southeastern Romania. Lancet.

[17] Lanciotti R.S., Roehrig J.T., Deubel V., Smith J., Parker M., Steele K. (1999). Origin of the West Nile virus responsible for an outbreak of encephalitis in the northeastern United States. Science.

[18] National Public Health Organisation (EODY) (2024). Annual reports about West Nile Virus (WNV) surveillance. https://eody.gov.gr/en/epidemiological-statistical-data/annual-epidemiological-data/.

[19] Tsioka K., Gewehr S., Pappa S., Kalaitzopoulou S., Stoikou K., Mourelatos S. (2023). West Nile virus in *Culex* mosquitoes in Central Macedonia, Greece, 2022. Viruses.

[20] European Centre for Disease Prevention and Control (ECDC) (2024). Annual reports about West Nile Virus (WNV) surveillance. https://www.ecdc.europa.eu/en/west-nile-fever/surveillance-and-disease-data/annual-epidemiological-report.

[21] Nasci R.S., Mutebi J.P. (2019). Reducing West Nile virus risk through vector management. J. Med. Entomol..

[22] Wimberly M.C., Davis J.K., Hildreth M.B., Clayton J.L. (2022). Integrated forecasts based on public health surveillance and meteorological data predict West Nile virus in a high-risk region of North America. Environ. Health Perspect..

[23] Giordano B.V., Kaur S., Hunter F.F. (2017). West Nile virus in Ontario, Canada: a twelve-year analysis of human case prevalence, mosquito surveillance, and climate data. PLoS One.

[24] Radojicic S., Zivulj A., Petrovic T., Nisavic J., Milicevic V., Sipetic-Grujicic S., Misic D., Korzeniowska M., Stanojevic S. (2021). Spatiotemporal analysis of West Nile virus epidemic in South Banat District, Serbia, 2017–2019. Animals.

[25] Sugumaran R., Larson S.R., DeGroote J.P. (2009). Spatio-temporal cluster analysis of county-based human West Nile virus incidence in the continental United States. Int. J. Health Geogr..

[26] Young SG, Jensen RR. Statistical and visual analysis of human West Nile virus infection in the United States, 1999–2008. Appl. Geogr., 34 (0) (2012), pp. 425–431, 10.1016/j.apgeog.2012.01.008.

[27] DeGroote J.P., Sugumaran R., Ecker M. (2014). Landscape, demographic and climatic associations with human West Nile virus occurrence regionally in 2012 in the United States of America. Faculty Publications..

[28] Rocheleau J.P., Kotchi S.O., Arsenault J. (2020). Can local risk of West Nile virus infection be predicted from previous cases? A descriptive study in Quebec, 2011–2016. Can. J. Public Health.

[29] Karki S., Brown W.M., Uelmen J., Ruiz M.O., Smith R.L. (2020). The drivers of West Nile virus human illness in the Chicago, Illinois, USA area: fine scale dynamic effects of weather, mosquito infection, social, and biological conditions. PLoS One.

[30] Chuang T.W., Hildreth M.B., Vanroekel D.L., Wimberly M.C. (2011). Weather and land cover influences on mosquito populations in Sioux Falls, South Dakota. J. Med. Entomol..

[31] Ferraguti M., Dimas Martins A., Artzy-Randrup Y. (2023). Quantifying the invasion risk of West Nile virus: insights from a multi-vector and multi-host SEIR model. One Health.

[32] Marini G., Manica M., Delucchi L., Pugliese A., Rosa R. (2021). Spring temperature shapes West Nile virus transmission in Europe. Acta Trop..

[33] Giesen C., Herrador Z., Fernandez-Martinez B., Figuerola J., Gangoso L., Vazquez A. (2023). A systematic review of environmental factors related to WNV circulation in European and Mediterranean countries. One Health.

[34] DeGroote J.P., Sugumaran R., Brend S.M., Tucker B.J., Bartholomay L.C. (2008). Landscape, demographic, entomological, and climatic associations with human disease incidence of West Nile virus in the state of Iowa, USA. Int. J. Health Geogr..

[35] Ferraccioli F., Riccetti N., Fasano A. (2023). Effects of climatic and environmental factors on mosquito population inferred from West Nile virus surveillance in Greece. Sci. Rep..

[36] Paz S., Malkinson D., Green M.S., Tsioni G., Papa A., Danis K. (2013). Permissive summer temperatures of the 2010 European West Nile fever upsurge. PLoS One.

[37] Stilianakis N.I., Syrris V., Petroliagkis T., Pärt P., Gewehr S., Kalaitzopoulou S. (2016). Identification of climatic factors affecting the epidemiology of human West Nile virus infections in northern Greece. PLoS One.

[38] Lorenz C., De Azevedo T.S., Chiaravalloti-Neto F. (2022). Impact of climate change on West Nile virus distribution in South America. Trans. R. Soc. Trop. Med. Hyg..

[39] Mavrakis A., Papavasileiou C., Alexakis D., Papakitsos E.C., Salvati L. (2021). Meteorological patterns and the evolution of West Nile virus in an environmentally stressed Mediterranean area. Environ. Monit. Assess..

[40] Tsioka K., Gewehr S., Kalaitzopoulou S., Pappa S., Stoikou K., Mourelatos S., Papa A. (2022). Detection and molecular characterization of West Nile virus in Culex pipiens mosquitoes in Central Macedonia, Greece, 2019-2021. Acta Trop..

[41] Reisen W.K., Lothrop H.D., Lothrop B.B. (2003). Factors influencing the outcome of mark-release-recapture studies with Culex tarsalis (Diptera: Culicidae). J. Med. Entomol..

[42] Verdonschot P.F.M., Besse-Lototskaya A.A. (2014). Flight distance of mosquitoes (Culicidae): a metadata analysis to support the management of barrier zones around rewetted and newly constructed wetlands. J. Vector Ecol..

[43] Hadjichristodoulou C., Pournaras S., Mavrouli M., Marka A., Tserkezou P., Baka A. (2015). West Nile virus Seroprevalence in the Greek population in 2013: a nationwide cross-sectional survey. PLoS One.

[44] Wang Y., Pons W., Fang J., Zhu H. (2017). The impact of weather and storm water management ponds on the transmission of West Nile virus. R. Soc. Open Sci..

[45] Reisen W.K., Fang Y., Martinez V.M. (2006). Effects of temperature on the transmission of west nile virus by Culex tarsalis (Diptera: Culicidae). J. Med. Entomol..

[46] O'brien, R.M (2007). A caution regarding rules of thumb for variance inflation factors. Qual. Quant..

[47] Moser S.K., Barnard M., Frantz R.M. (2023). Scoping review of Culex mosquito life history trait heterogeneity in response to temperature. Parasit. Vectors.

[48] Albrecht L., Kaufeld K.A. (2023). Investigating the impact of environmental factors on West Nile virus human case prediction in Ontario. Canada. Frontiers in public health.

