# Supplementary material for: Unveiling spatial patterns of West Nile virus emergence in northern Greece, 2010–2023
Source: One Health. 2024 Sep 5;19:100888. doi: 10.1016/j.onehlt.2024.100888 (PMC11406245; doi:10.1016/j.onehlt.2024.100888)
Supplement: Supplementary file 1 — Supplementary material. [file mmc1.pdf]

S1 Appendix – General information about Region of Central Macedonia

Investigation area

According to the 2021 census, the region has a population of 1,792,069 inhabitants and covers an area of 18,811 km^2. RCM is divided into seven provinces and 38 municipalities. The municipalities and the ID number of each municipality in RCM are presented in Fig S1 and Table S1. There is an additional province called "Mount Athos" (colored white in Fig S1), it is a self-governing monastic community and is not included in this research.

WNV in RCM

RCM in Northern Greece is an area of major epidemiological interest for the study of WNV due to the increased number of human cases recorded since 2010. Outside the WNV hiatus period in RCM (2014-2017), the percentage of municipalities affected out of the total number of municipalities in this region varied from 26% (2012) to 82% (2010), while the percentage of the number of WNVhc in this region in relation to the total number of WNVhc in Greece varied between 13% (2012) and 95% (2010). In the period 2010-2023, 51% of the total WNVhc in Greece were recorded in the RCM, and 24% of the total number of affected municipalities were observed in this region.

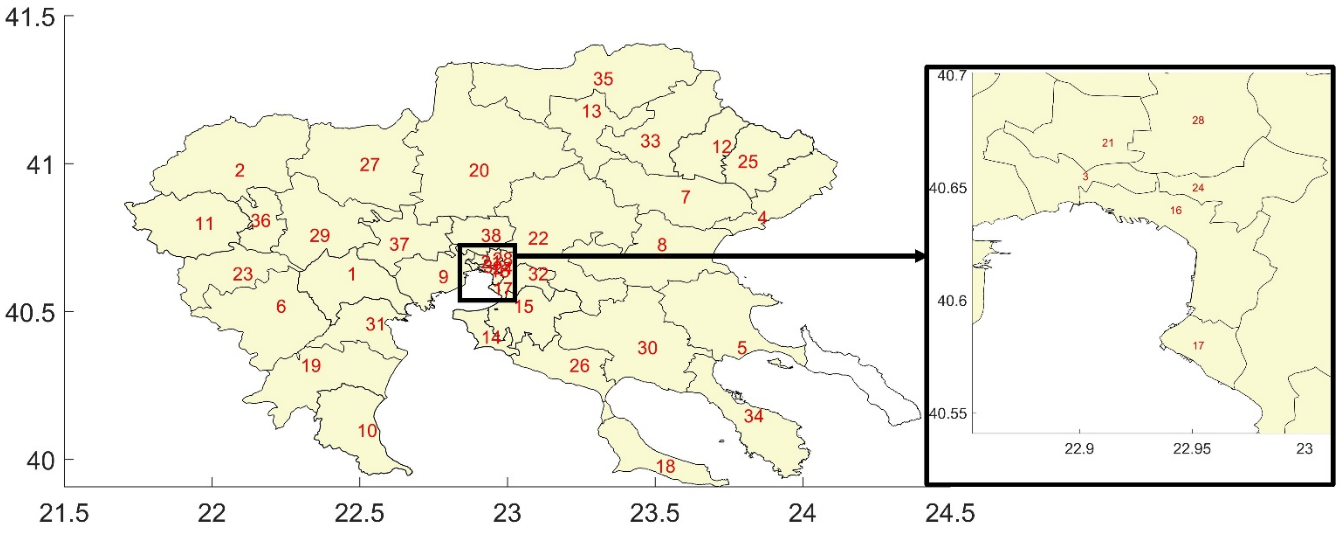

Fig S1. Map of the municipalities of the region included in the study. The ID number of each municipality is given on the map. Some urban municipalities of the region are quite close, so the right map focuses on them.

Table S1. Municipalities of the RCM.

Municipality ID	Municipality Name	Municipality ID	Municipality Name
1	ALEXANDRIA	20	KILKIS
2	ALMOPIA	21	KORDELIO-EVOSMOS
3	AMPELOKIPOI-MENEMENI	22	LAGADAS
4	AMFIPOLIS	23	NAOUSA
5	ARISTOTELIS	24	NEAPOLI-SIKES
6	VERIA	25	NEA ZIXNI
7	VISALTIA	26	NEA PROPONTIDA
8	VOLVI	27	PAIONIA
9	DELTA	28	PAVLOS MELAS
10	DION-OLIMPOS	29	PELLA
11	EDESSA	30	POLIGIROS
12	EMMANOUIL PAPPAS	31	PYDNA KOLINDROS
13	IRAKLIA	32	PILEA-HORTIATI
14	THERMAIKOS	33	SERRES
15	THERMI	34	SITHONIA

16	THESSALONIKI	35	SINTIKI
17	KALAMARIA	36	SKIDRA
18	KASSANDRA	37	HALKIDONA
19	KATERINI	38	OREOKASTRO

Land cover and elevation data

Information on land cover and elevation data are in the coordination of information on the environment (CORINE) system. CORINE Land Cover (CLC) is a digital mapping and inventory system that provides detailed and standardized information on land cover and land use across Europe, while data were obtained through the interpretation of satellite images and aerial photographs, combined with other geospatial data sources. Figs S2A and S2B show the land use type of each municipality and the geomorphology of the municipalities. Analytical study of land use type and altitude is necessary to understand and interpret the results. Maps were elaborated in the ArcGIS software.

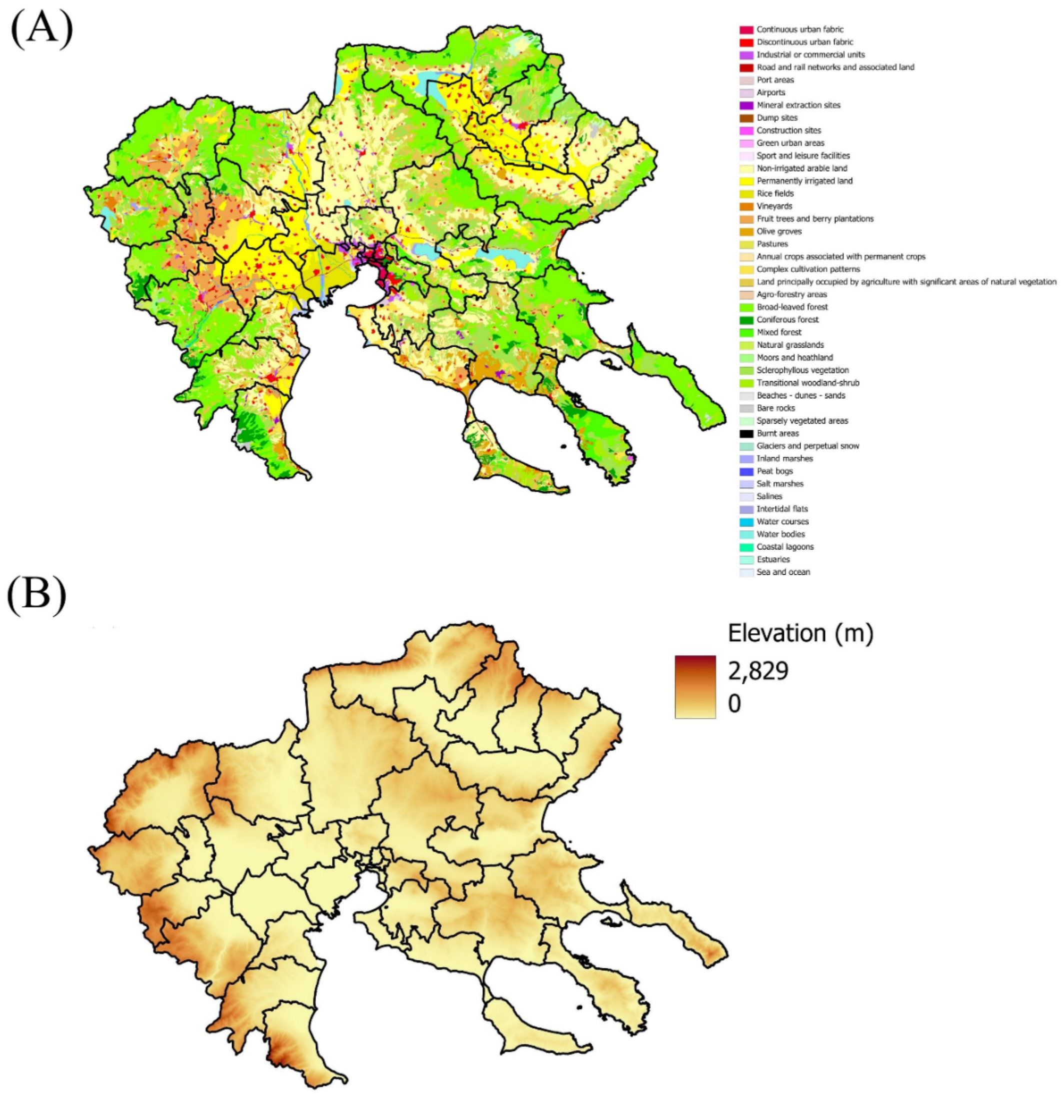

Fig S2. Maps of (A) the land use distribution in each municipality split into 44 classes and (B) the elevation distribution in each municipality.

S2 Appendix – Investigation of Culex measurements

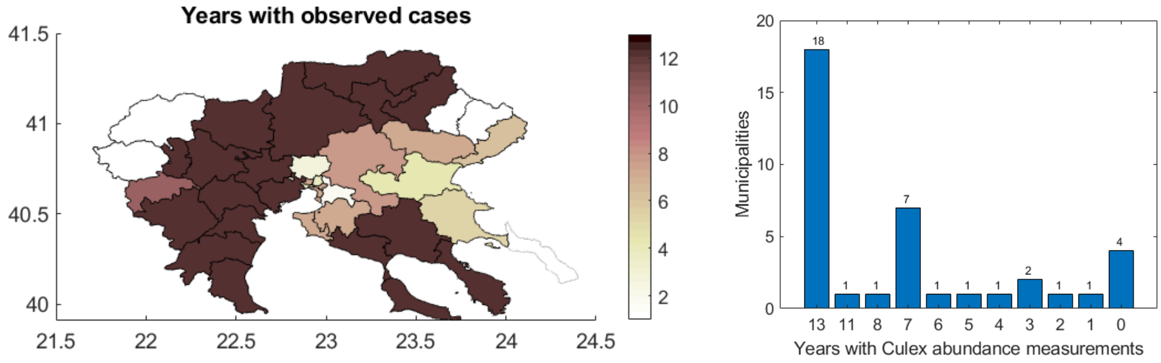

Fig S3. Geographical distribution of the number of years per municipality with Culex abundance measurements over the period 2011–2023.

Table S2. The month of first and last sampling each year in the municipalities of the RCM.

Month	Year												
	2011	2012	2013	2014	2015	2016	2017	2018	2019	2020	2021	2022	2023
of first sampling	4	5	6	5	6	5	5	4	4	5	5	4	4
of last sampling	9	10	9	10	9	9	9	9	9	9	9	9	10

Table S3. Number of months with (monthly) mosquito abundance measurements were recorded per year in each municipality.

Number of monthly measurements of Culex per year													
Municipalities	2011	2012	2013	2014	2015	2016	2017	2018	2019	2020	2021	2022	2023
1	6	6	4	5	4	4	5	6	5	5	5	6	6
2	0	0	0	0	0	0	0	0	0	0	0	0	0
3	0	0	0	0	0	0	0	0	0	0	3	5	6
4	0	2	3	4	4	3	0	0	0	1	0	0	0
5	0	2	3	4	4	3	0	0	0	0	0	0	0
6	4	4	3	4	4	3	5	5	5	5	5	5	6
7	0	0	0	0	0	0	3	5	5	5	4	6	5
8	0	3	0	0	0	0	5	4	0	1	0	0	2
9	6	6	4	6	4	5	5	6	6	5	5	6	6
10	3	5	4	5	4	4	5	6	5	5	5	5	7
11	0	0	0	0	0	0	0	0	0	0	0	0	0
12	0	0	0	0	0	0	0	0	0	0	0	0	0
13	5	6	4	5	4	3	5	5	5	5	4	5	5
14	0	0	0	0	0	0	5	6	5	5	5	5	6
15	0	0	0	0	0	0	5	6	5	5	5	6	6
16	0	0	0	0	0	0	5	6	5	5	5	6	6
17	0	0	0	0	0	0	5	6	5	5	5	6	6
18	5	6	4	5	4	4	4	6	5	5	5	5	5
19	4	5	4	5	4	4	5	6	5	5	5	5	7
20	6	6	4	5	4	5	5	5	5	5	5	5	5
21	0	0	0	0	0	0	5	4	4	5	5	5	6
22	0	3	0	0	0	0	5	6	5	5	5	5	5
23	5	4	3	0	0	3	4	5	5	5	4	5	6
24	0	0	0	0	0	0	4	5	5	5	4	6	5
25	0	0	0	0	0	0	0	0	0	0	0	0	0
26	5	6	4	5	4	4	5	6	5	5	5	5	5
27	6	6	4	5	4	5	5	5	5	5	5	5	5
28	0	0	0	0	0	0	0	0	0	0	3	5	5
29	5	6	4	5	4	5	5	5	5	5	5	6	6
30	4	4	3	4	4	3	4	5	5	4	5	5	5
31	6	6	4	5	4	4	5	6	5	5	5	5	7
32	0	0	0	0	0	0	0	0	0	0	0	1	1
33	5	6	4	5	4	5	5	5	5	5	5	6	5
34	3	4	3	4	4	3	1	5	5	4	5	5	5
35	4	4	3	4	4	3	5	5	5	5	4	6	5
36	5	6	4	5	4	4	5	5	5	5	5	5	6
37	5	6	4	6	4	5	5	6	5	5	5	5	5
38	0	0	0	0	0	0	5	0	0	0	0	1	0

S3 Appendix – Study of WNV incidence data

Laboratory-confirmed WNVhcP100TH

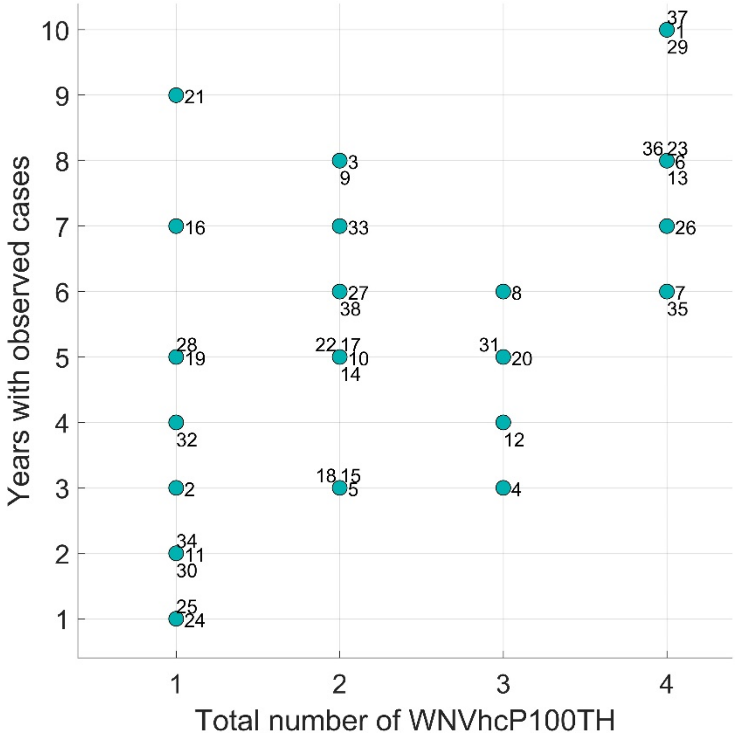

Fig S4. Scatter plot of years with observed cases VS total number of WNVhcP100TH (i.e. the sum of all cases per 100,000 population over 2010-2023) (1: 1≤ cases<25, 2: 25≤cases<50, 3: 50≤cases<75 and 4: cases≥75). Every point represents different municipality/ies, while the number of each point corresponds to the ID number of each municipality.

Table S4. Total annual number of WNVhcP100TH in all municipalities of the region.

Year	Total number of WNVhcP100TH
2010	594
2011	84
2012	44
2013	62
2018	269
2019	149
2020	350
2021	113
2022	502
2023	188

S4 Appendix – Global Moran's I Index

Table S5. Yearly Global Moran's I indices of MCPL, MIMS, WNVhcP100TH. Bold values correspond to the significance level of 0.01, while values with * correspond to the significance level of 0.001.

Year	MCPL	MIMS	WNVhcP100TH
2010	-	-	**0.256**
2011	**0.711***	0.216	0.138
2012	**0.613**	-0.262	-0.005
2013	**0.547**	0.338	0.068
2014	**0.461**	-	-
2015	**0.669***	-	-
2016	**0.352**	-	-
2017	**0.527**	-	-
2018	**0.527**	0.082	**0.339**
2019	0.444	-0.1	0.155
2020	**0.479**	**0.594**	**0.503**
2021	-0.029	0.03	**0.271**
2022	**0.452**	0.329	0.041
2023	**0.622***	0.287	0.039
Median (years without measurements are excluded)	**0.626**	**0.478**	**0.319**

S5 Appendix – Focus on Generalized Linear Mixed Models

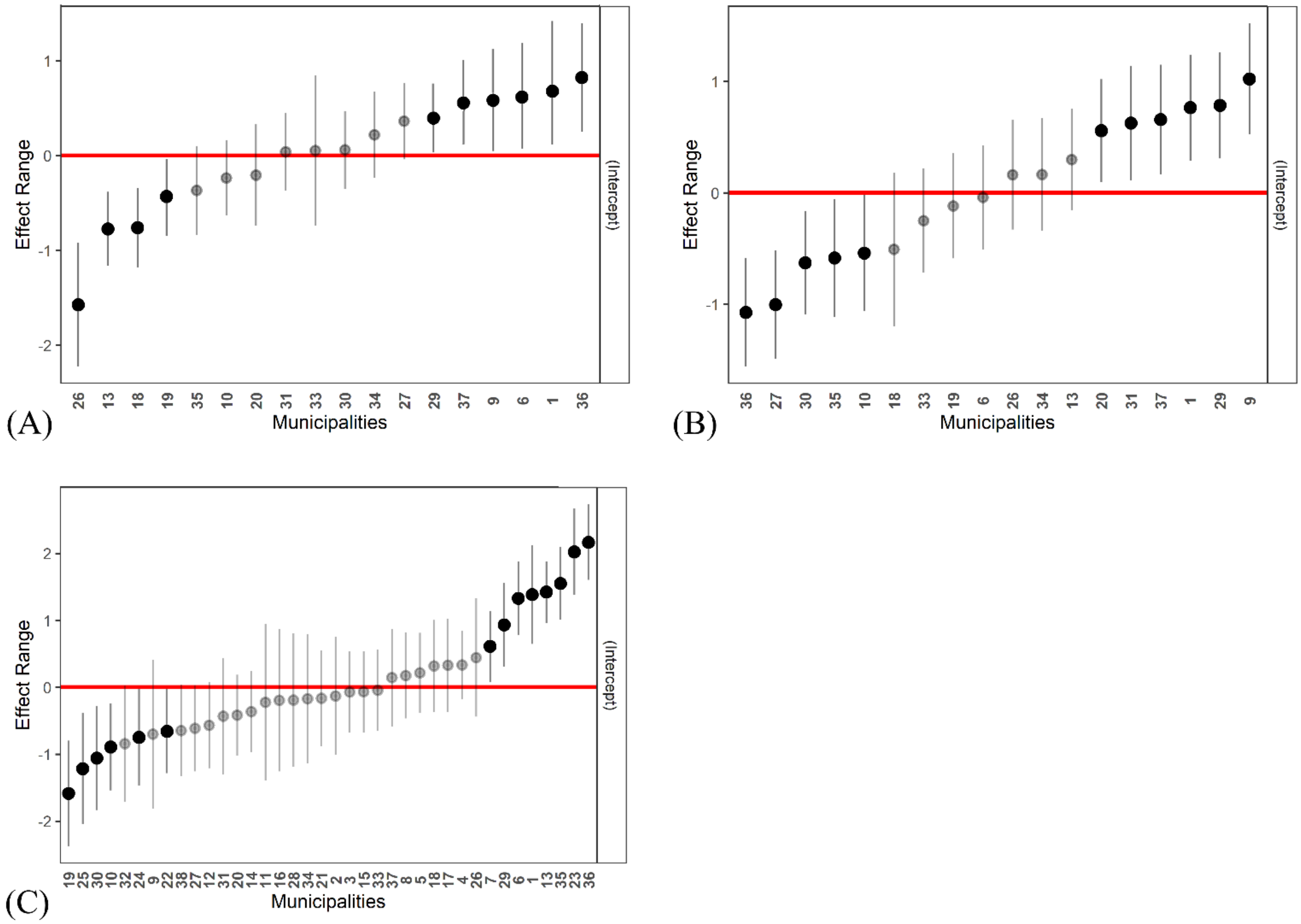

Fig S5. Random effect range derived from a GLMM to estimate the influence of seasonal temperature anomalies to the (A) MCPL, (B) MIMS and (C) WNVhcP100TH.

Table S6. Training and prediction NMAE for different years. For each year, the model is trained using all years except the year used for prediction. Dashed years (-) excluded from the research.

Year	MCPL		MIMS		WNVhcP100TH	
	Train	Test	Train	Test	Train	Test
2011	0.36	0.37	1.00	0.97	0.69	1.33
2012	0.36	0.38	1.04	0.90	0.68	2.80
2013	0.36	0.32	1.02	1.49	0.71	0.84
2014	0.34	0.48	-	-	-	-
2015	0.37	0.35	-	-	-	-
2016	0.36	0.31	-	-	-	-
2017	0.37	0.23	-	-	-	-
2018	0.34	1.18	0.96	6.89	0.72	0.89
2019	0.35	0.47	0.94	17.58	0.70	0.92
2020	0.36	0.40	1.04	1.05	0.68	0.91
2021	0.35	0.57	0.98	1.21	0.69	1.26
2022	0.35	0.47	0.89	1.00	0.73	0.80
2023	0.35	0.56	1.05	0.92	0.70	0.66

Fig S6, representing actual values, provides a good justification for the outliers identified in Fig 5A. Specifically, significant deviations in the actual average yearly values (shown by the blue line in Fig S6) compared to the average excluding that year (shown by the red line in Fig S6) correspond well with the outliers marked in Fig 5A, supporting the conclusion that these outliers are due to extreme actual values in those years.

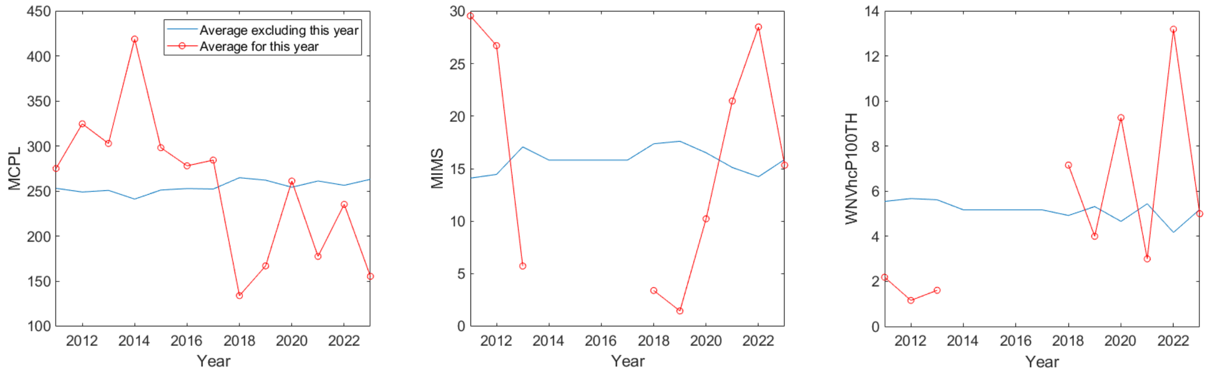

Fig S6. The red line represents the the timeseries of actual average yearly values for MCPL, MIMS and WNVhcP100TH, while the blue line represents the the timeseries of average values excluding this year.

The geographic distribution of the median of predicted and observed values for each variable (Fig S7) combined with the geographical distribution of NMAE (Fig 5B) provide a clear and comprehensive overview of the model's performance across different municipalities of the region.

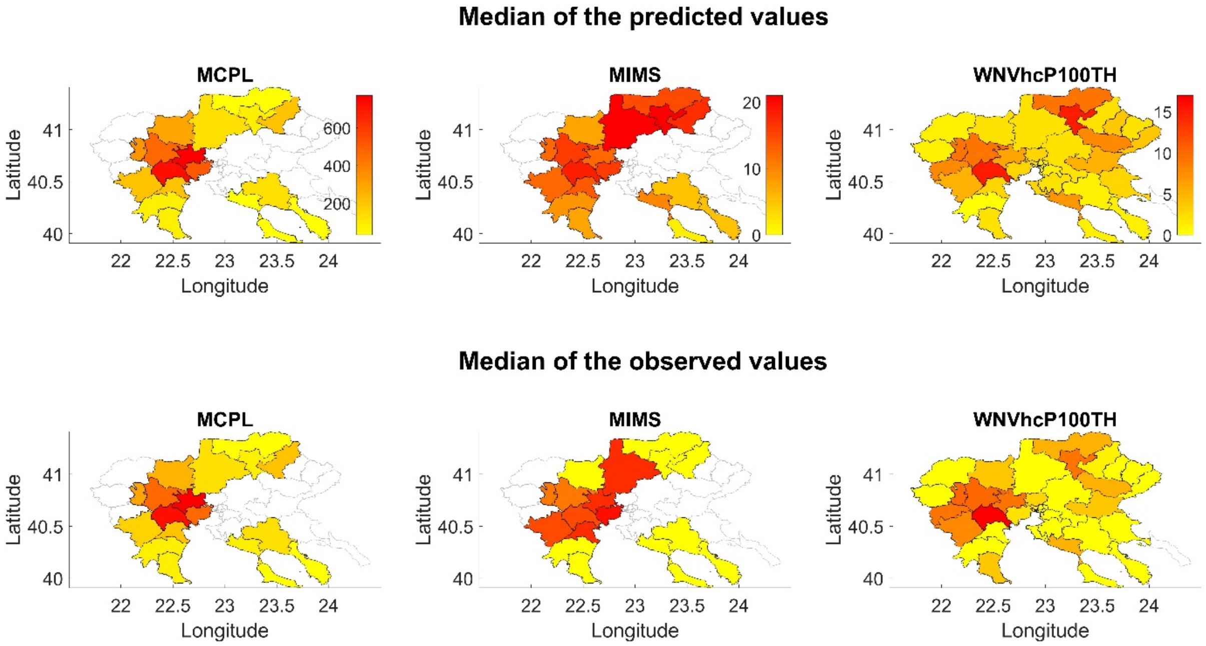

Fig S7. Maps of medians of predicted (top) and observed (bottom) MCPL, MIMS, WNVhcP100TH in each municipality. The predicted values at each municipality corresponds to the median between all-years through a leave one-year-out cross validation approach.
